# Evaluation of different isotope dilution mass spectrometry strategies for the characterization of naturally abundant and isotopically labelled peptide standards

**DOI:** 10.1007/s00216-024-05176-1

**Published:** 2024-02-16

**Authors:** Jesús Nicolás Carcelén, Helí Potes Rodríguez, Adriana González-Gago, Juan Manuel Marchante-Gayón, Alfredo Ballesteros, José Manuel González, José Ignacio García Alonso, Pablo Rodríguez-González

**Affiliations:** 1 Department of Physical and Analytical Chemistry, Faculty of Chemistry, University of Oviedo, Oviedo, Spain; 2 Department of Organic and Inorganic Chemistry, Faculty of Chemistry, University of Oviedo, Oviedo, Spain

**Keywords:** Isotope dilution analysis, Mass spectrometry, Peptide characterization

## Abstract

Natural abundance and isotopically labelled tryptic peptides are routinely employed as standards in quantitative proteomics. The certification of the peptide content is usually carried out by amino acid analysis using isotope dilution mass spectrometry (IDMS) after the acid hydrolysis of the peptide. For the validation and traceability of the amino acid analysis procedure, expensive certified peptides must be employed. In this work we evaluate different IDMS alternatives which will reduce the amount of certified peptide required for validation of the amino acid analysis procedure. In this context, the characterization of both natural and isotopically labelled synthetic angiotensin I peptides was carried out. First, we applied a fast procedure for peptide hydrolysis based on microwave-assisted digestion and employed two certified peptide reference materials SRM 998 angiotensin I and CRM 6901-b C-peptide for validation of the hydrolysis procedure. The amino acids proline, leucine, isoleucine, valine, tyrosine, arginine and phenylalanine were evaluated for their suitability for peptide certification by IDMS by both liquid chromatography with tandem mass spectrometry (LC–MS/MS) and gas chromatography with mass spectrometry (GC)–MS/MS. Then, natural angiotensin I and ^13^C_1_-labelled angiotensin I were synthesized in-house and purified by preparative liquid chromatography. The concentration of the ^13^C_1_-labelled angiotensin I peptide was established by reverse IDMS in its native form using SRM 998 angiotensin I as reference. The concentration of the natural synthesized peptide was determined by IDMS both using the ^13^C_1_-labelled peptide in its native form and by amino acid analysis showing comparable results. Finally, the synthetic naturally abundant angiotensin I peptide was employed as “in-house” standard for the validation of subsequent peptide characterization procedures. Therefore, the novelty of this work relies on, first, the development of a faster hydrolysis procedure assisted by focused microwaves, providing complete hydrolysis in 150 min, and secondly, a validation strategy combining GC–MS and LC–MS/MS that allowed us to certify the purity of an in-house-synthesized peptide standard that can be employed as quality control in further experiments.

**Graphical abstract:**

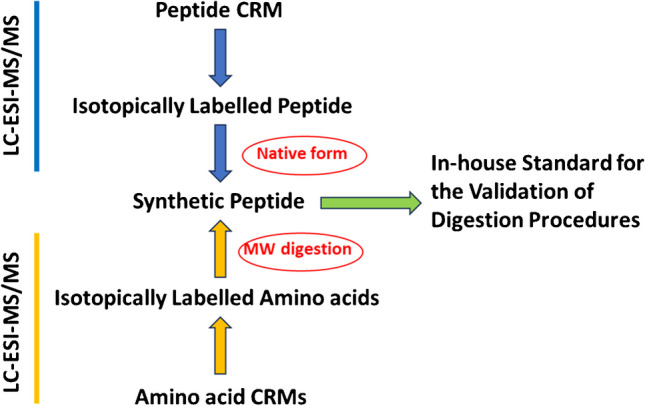

**Supplementary Information:**

The online version contains supplementary material available at 10.1007/s00216-024-05176-1.

## Introduction

The concentration of certain proteins and peptides in biological fluids may play an important role in the early detection, diagnosis, monitoring and treatment of certain diseases. For example, angiotensin has been shown to be a biomarker for hypertension, while insulin and C-peptides have been considered biomarkers for diabetes. In this context, new candidate protein/peptide biomarkers are continuously being proposed for the early detection of diseases [1]. However, for the approval of validated biomarkers by regulatory agencies, metrological tools for their accurate quantification need to be developed. Unfortunately, the availability of reference measurement procedures, pure substance standards and matrix-certified reference materials are still very limited for protein and peptide analysis [2]. The mass balance method [3] is not well suited for certification in the case of peptides and proteins because only small amounts of material are usually available, and thus alternative procedures need to be developed.

Isotope dilution in combination with mass spectrometry (IDMS) has been considered a primary method of measurement for the unit “amount of substance”. The amount of substance of a system is defined by IUPAC as a measure of the number of specified elementary entities, where an elementary entity is an atom, a molecule, an ion, an electron, or any other particle or specified group of particles. IDMS is able to provide results directly traceable to the International System of Units (SI) [4], and is thus a highly appropriate method for the absolute quantification of proteins and peptides. In the case of proteins, the absolute quantification strategy is usually based on the determination of “proteotypic” peptides (those peptides describing a sequence that is only found in a single known protein and therefore serves to identify and confirm its presence in a sample) by IDMS after the quantitative enzymatic digestion of the proteins and the use of isotopically labelled peptides. This strategy is able to produce highly accurate and precise analytical data [5].

Nowadays, the commercial availability of “proteotypic” peptides is huge, and many companies are able to synthesize customized natural and isotope-labelled peptides to be applied to each analytical problem. The main problem is that such commercial standards are very often lacking from an SI traceable purity certification, which is essential for the reliable quantification of the target protein. Therefore, an in-house certification of the standards is usually required to ensure the quality and comparability of the analytical results.

According to the literature, there are several approaches that can be used to assess the purity of peptide primary standards [6]. The procedure based on amino acid analysis (AAA) of the “proteotypic” peptide by IDMS quantification of the released amino acids after complete hydrolysis is frequently chosen for this purpose [7]. The hydrolysis of a peptide can be carried out by following a chemical or enzymatic procedure, although the enzymatic approach is seldom used, as several enzymes are required for a complete digestion, and the complete hydrolysis usually takes longer. The chemical hydrolysis consists of the addition of a hydrolysis agent and incubation of the mixture under controlled working conditions (temperature, time, concentration and type of hydrolysis agent and additives). The heating of the solutions with traditional equipment such as oil baths, sand baths or heating blankets not only is slow, but creates a hot surface on the reaction vessel in which analytes and reagents could eventually decompose. Alternatively, microwave heating in a well-designed container allows a homogeneous increase in the temperature throughout the sample, leading to less analyte and reagent decomposition [8]. In addition, microwaves reduce energy consumption, shorten the reaction time, prevent overheating of the surfaces, allow high chemical reaction yields, reduce the formation of secondary products, eliminate the restriction of the boiling points and are environmentally friendly due to the use of lower volumes of acids and solvents [9]. This technology has been used to accelerate reactions such as peptide synthesis, enzymatic digestion and acid hydrolysis, applying high temperatures for short periods of time. Commercial focused microwave systems designed specifically to perform extractions of chemical compounds allow reproducible and uniform irradiation in all samples [10]. However, acid hydrolysis of peptide standards to assess their purity by IDMS quantification of the released amino acids has not yet been systematically studied [11]. Additionally, the validation and traceability of acid hydrolysis procedures require the use of expensive certified reference materials, and thus alternative procedures for validation need to be developed.

The aim of this work is to compare two alternative IDMS methods for the certification of peptide standards: AAA after acid hydrolysis and direct determination of the peptide without hydrolysis. This requires the development and validation of a hydrolysis procedure for AAA which is carried out here by applying focused microwaves to decrease the total hydrolysis time applied in comparison with classical procedures. In this way, a traceable procedure can be developed which employs cheaper synthetic peptide standards. We took advantage of the existence of an angiotensin I certified reference material for this purpose. Thus, we synthesized the angiotensin I peptide, both in its natural abundance form and in the form of a minimally ^13^C_1_-labelled analogue. The reference material was employed for the optimization of the peptide hydrolysis method, based on microwave digestion, and for the certification of the ^13^C_1_-labelled angiotensin I peptide. The purity of the synthesized natural angiotensin I peptide was then determined both by AAA and by direct IDMS determination using the ^13^C_1_-labelled peptide.

## Experimental

### Reagents and materials

Wang-type resins preloaded with fluorenylmethoxycarbonyl (Fmoc)-protected Leu (extent of labelling: 0.65 mmol/g loading) from Sigma-Aldrich (St. Louis, MO, USA) were used for the solid-phase synthesis of natural and labelled peptides. Individual Fmoc-protected amino acids (Asp, Arg, Val, Tyr, Ile, His, Pro, Phe) from Sigma-Aldrich or Merck (Darmstadt, Germany) were used to grow the peptide chain. Labelled (Fmoc)Val (^13^C_1_, 99.2%) from Cambridge Isotope Laboratories was used for the synthesis of the labelled peptides. Other reagents used in the synthesis reaction included ethyl-2-cyano-2-(hydroxyimino)acetate (oxyma), *N*-hydroxybenzotriazole (HOBt), *N*,*N*′-diisopropylcarbodiimide (DIC), triisopropylsilane (TIS), piperazine, ethanol, *N*-methylpyrrolidone (NMP), trifluoroacetic acid (TFA), dichloromethane and diethyl ether from Sigma-Aldrich and *N*,*N*-dimethylformamide (DMF) from BDH Prolabo (Leuven, Netherlands).

Certified reference materials (TraceCERT^®^) of the amino acids L-Pro (99.8 ± 0.2%), L-Leu (99.5 ± 0.1%), L-Ile (98.9 ± 0.2%), L-Val (99.4 ± 0.1%), L-Tyr (99.5 ± 0.1%), L-Arg (99.3 ± 0.1%) and L-Phe (99.7 ± 0.1%) were obtained from Fluka Analytical (Munich, Germany). Isotopically labelled L-Pro-^13^C_1_ (≥ 99%), L-Leu-^13^C_1_ (≥ 99%), L-Ile-^13^C_1_ (≥ 98%), L-Val-^13^C_1_ (≥ 99%), L-Phe-^13^C_1_ (≥ 99%) and L-Arg-^15^N_4_ (≥ 98%) were purchased from Sigma-Aldrich. Isotopically labelled L-Tyr-^13^C_2_ (positions 3 and 5 in the aromatic ring, 99%) was purchased from Cambridge Isotope Laboratories. Additionally, a certified reference material (TraceCERT^®^) containing a mixture of 17 proteinogenic amino acids was purchased from Sigma-Aldrich. Butanol/HCl 3 M (99.9%) and *N*-methyl-*N*-(tert-butyldimethylsilyl) trifluoroacetamide were used as amino acid derivatizing reagent and stored in a refrigerator at 4 °C.

In the preparation of the mobile phases, methanol of high purity (≥ 99.9%) was purchased from Fisher Scientific (Waltham, MA, USA) and formic acid (> 98%) and trifluoroacetic acid (TFA) from Sigma-Aldrich.

Two peptide reference materials were used for the validation of the hydrolysis procedure. SRM 998 angiotensin I (human) was purchased from the National Institute of Standards and Technology (NIST), whereas CRM 6901-b C-peptide was purchased from the National Metrology Institute of Japan. For the hydrolysis of the peptides, 12 M HCl purified by means of a sub-boiling distillation process was used. A DST-1000 acid distiller (Savillex, LLC, Eden Prairie, MN, USA) was also employed.

### Instrumentation

A high-performance liquid chromatography (HPLC) system (Agilent 1260 Infinity, Agilent Technologies, Santa Clara, CA, USA) equipped with an analytical-scale fraction collector and a variable-wavelength detector was employed for the purification of the synthetic peptide by semi-preparative liquid chromatography. A reversed-phase column (Aeris Peptide XB-C18, 250 × 4.6 mm, 5 μm particle size and 100 Å pore size) from Phenomenex (Torrance, CA, USA) was used for this purpose.

An Agilent 1290 Series liquid chromatograph (Agilent Technologies, Santa Clara, CA, USA) coupled to an Agilent 6460 triple quadrupole mass spectrometer equipped with an electrospray ionization (ESI) interface with jet stream operating in positive ion mode was employed both for the direct analysis of the peptides and for the analysis of the derivatized amino acids. In both cases a ZORBAX Eclipse Plus C18 (2.1 × 50 mm; 1.8 μm) column (Agilent Technologies) was used.

A gas chromatograph (Agilent 7890A, Agilent Technologies, Santa Clara, CA, USA) fitted with a split/splitless injector and a DB-5ms capillary column (30 m × 0.25 mm i.d., 0.25 μm film thickness, Agilent Technologies) coupled to an Agilent 7000 triple quadrupole mass spectrometer with an electron ionization (EI) source was used for the determination of derivatized amino acids.

All solutions were prepared by the gravimetric method on an analytical balance (AB204-S, Mettler Toledo, Columbus, Ohio, USA). All solutions were homogenized using a vortex shaker (VELP Scientifica, Usmate Velate, Italy). In the derivatization process, a Micro Star 17 centrifuge (VWR, Radnor, PA, USA) and a miVac evaporator from Supelco (St. Louis, MO, USA) were used to evaporate the solvent, and a Heraeus^®^ oven (Thermo Scientific, Waltham, MA, USA) was used for the esterification reaction.

The peptide hydrolysis process was carried out in a Biotage Initiator™ (Biotae, Uppsala, Sweden) focused microwave oven. A Liberty Blue™ automated microwave peptide synthesizer (CEM Corporation, Matthews, NC, USA) was used for the synthesis of natural and labelled peptides.

Ultrapure water (18.2 MΩ m) was obtained from a Milli-Q Gradient A10 purification system (MilliporeSigma, Burlington, MA, USA). A BASIC 20 pH meter (Crison Instruments, Barcelona, Spain) was also used. Classical tubes for vacuum hydrolysis with a volume of 6 mL were purchased from Thermo Scientific, and tubes for focalized microwave hydrolysis with a volume of 2 mL were purchased form Biotage (Sweden).

### Procedures

#### Natural abundance and labelled amino acid derivatization

Two different amino acid derivatization procedures were carried out, depending on the chromatographic separation. For reversed-phase LC separation, a Fisher esterification using butanol-HCl was employed to improve the separation and detection of the amino acids [12]. Briefly, solutions containing amino acids were placed in Eppendorf^®^ tubes and evaporated under vacuum. Then, 100 µL of butanol-HCl 3 M was added and heated in a thermal block (with agitation) at 60 °C for 30 min. This solution was then evaporated under vacuum and the dry residue was reconstituted in the initial mobile phase and subjected to analysis.

Regarding gas chromatography (GC) separation, volatile derivatives were obtained through a silanization reaction with *N*-methyl-*N*-(tert-butyldimethylsilyl)trifluoroacetamide (MTBSTFA) with 1% tert-butyldimethylchlorosilane (TBDMS) [13]. Briefly, solutions containing amino acids were placed in Eppendorf^®^ tubes and evaporated under vacuum. Then, 50 µL of pyridine was added and stirred for 15 min. Next, 50 µL of MTBSTFA with 1% TBDMS was added, and the mixture was shaken and heated in a thermal block at 60 °C for 30 min. This solution was directly injected into the instrument. A scheme for the LC and GC derivatization reactions is presented in Figure S1 of the Supporting Information.

#### LC–MS/MS analysis

The chromatographic separation of arginine, proline, valine, tyrosine, isoleucine, leucine and phenylalanine was carried out as butyl ester derivatives following a procedure described elsewhere [14].The experimental chromatographic conditions are shown in Table S1 of the Supporting Information. The same chromatographic conditions were employed for the separation of angiotensin I from its impurities. MS/MS analysis was performed using positive-ion ESI and selected reaction monitoring (SRM) mode. Direct injections of 1 µg g^−1^ standard solutions of natural abundance amino acids dissolved in a mixture of mobile phases A and B (1:1) were performed for the optimization of instrumental parameters. The identification of amino acids was carried out by LC–MS based on the retention time and the related m/z values of individual standards. An Agilent MassHunter Workstation (version B.06.00) was used for data acquisition and treatment. Experimental mass spectrometric conditions are summarized in Table S1 of the Supporting Information.

#### GC–MS/MS analysis

The chromatographic separation of proline, valine, tyrosine, isoleucine, leucine and phenylalanine was carried out as TBDMS derivatives using a DB-5ms column (30 m × 0.25 mm and 0.25 µm particle size). Helium was used as carrier gas with a linear flow of 2 mL/min. The identification of the amino acids was performed by comparison of the experimental spectrum in SCAN mode with Wiley and NIST MS libraries. MS/MS analysis was performed using SRM mode. The data acquisition and treatment were carried out using an Agilent MassHunter Workstation (version B.06.00). The experimental chromatographic and mass spectrometric conditions are summarized in Table S2 of the Supporting Information.

#### Acid hydrolysis of peptides for amino acid analysis (AAA)

All peptide standard solutions stored at −20 °C were thawed and homogenized by vigorous shaking using a vortex. Solutions containing amino acids stored at 4 °C were allowed to reach room temperature. Then, ca. 50 mg of a 100 µg g^−1^ solution of peptide and 100 mg of a mixture of labelled amino acids were added. The concentration of the labelled amino acids in the solution ranged from 10 to 40 µg g^−1^ depending on the initial concentration of the peptide standard to be characterized. The addition of sample and labelled standards was always controlled by gravimetric measurement. Subsequently, ca. 150 µL of H_2_O and 300 µL of sub-boiling HCl 12 M were pipetted into the glass vials. Finally, vials were sealed and the air inside was exchanged with N_2_ to avoid oxidation during the microwave hydrolysis. Then the vials were introduced into a focused microwave Biotage Initiator to carry out the peptide hydrolysis for 150 min at 150 °C. Figure S2 in the Supporting Information depicts this procedure graphically.

#### Synthesis of labelled and natural abundance angiotensin I

Natural and ^13^C_1_-labelled angiotensin I were synthesized following a solid-phase peptide synthesis (SPPS) procedure [15]. Briefly, amino acid residues were coupled sequentially to a solid support (polystyrene beads) from C-terminus to N-terminus. Amino acids with temporary protecting groups in the amino terminus and permanent protecting groups in the side chain were used. The coupling of the incoming amino acid to the growing chain was performed by a three-stage cyclic procedure. First, the N-terminus of the last amino acid in the peptide chain was deprotected, then the incoming amino acid (carboxyl terminus) was coupled to the peptide chain (amino terminus), and finally the excess of the reagents was removed by washing. Deprotection and activation solutions and additives were used to improve the efficiency of the coupling reaction as well as minimizing side reactions. The SPPS was performed using an automated microwave peptide synthesizer.

Once the desired peptide was obtained as a chain bound to the solid support, it was filtered and washed. Then the solid residue was treated with a mixture of TFA, TIS and ultra-pure water (95:2.5:2.5) to remove all the permanent protecting groups from the side chains and to cleave the peptide from the solid support. Afterwards, the solution was filtered, and the peptide was precipitated with iced diethyl ether. After several cycles of centrifugation and cleaning with iced diethyl ether, the obtained solid was freeze-dried and stored in the dark at −20 °C.

The purification of the synthesized peptides was carried out by semi-preparative HPLC as described in Table S3 of the Supporting Information. In brief, 10 mg of the solid was dissolved in 1 mL H_2_O and 0.1% (v/v) TFA, and 50 µL of the solution was injected per run. Figure S3 in the Supporting Information shows the experimental design of this work. It is worth noting that the amount of purified peptide synthesized in house ranged from 10 to 100 mg, which is a much larger amount than that provided in the reference materials NIST 998 angiotensin I (ca. €1000/0.5 mg) and CRM 6901-b C-peptide purchased from the National Metrology Institute of Japan (ca. €1000/ 0.1 mg).

#### IDMS calculations

The developed measurement methods were used with an isotope pattern deconvolution (IPD) approach to perform quantification. This strategy, which was published previously by our group [16], allows for overlapping spectra between natural and labelled compounds and does not require the construction of a methodological calibration graph. Briefly, for each compound, a two-pattern Eq. (1) was employed:

1 $$\left[\begin{array}{c}\begin{array}{c}{A}_{1}^{m}\\ {A}_{2}^{m}\\ \dots \end{array}\\ \begin{array}{c}\dots \\ {A}_{n-1}^{m}\\ {A}_{n}^{m}\end{array}\end{array}\right]=\left[\begin{array}{cc}\begin{array}{c}{A}_{1}^{s}\\ {A}_{2}^{s}\\ \dots \end{array}& \begin{array}{c}{A}_{1}^{t}\\ {A}_{2}^{t}\\ \dots \end{array}\\ \begin{array}{c}\dots \\ {A}_{n-1}^{s}\\ {A}_{n}^{s}\end{array}& \begin{array}{c}\dots \\ {A}_{n-1}^{t}\\ {A}_{n}^{t}\end{array}\end{array}\right]\times \left[\begin{array}{c}{x}_{s}\\ {x}_{t}\end{array}\right]+\left[\begin{array}{c}\begin{array}{c}{e}_{1}\\ {e}_{2}\\ \dots \end{array}\\ \begin{array}{c}\dots \\ {e}_{n-1}\\ {e}_{n}\end{array}\end{array}\right]$$

where *A*^*m*^, *A*^*s*^ and *A*^*t*^ are the relative abundances for *n* selected nominal masses for the mixture (*m*), the sample (*s*) and the tracer (*t*), respectively. Furthermore, unknowns *x*_*s*_ and *x*_*t*_, which represent the molar fractions of sample and tracer in the mixture, are calculated by a simple matrix inversion procedure. For the IDMS determination of the studied compounds, typically four masses of the most abundant isotopologues from the natural and labelled compounds were used.

Once the molar fractions were calculated, the final concentrations of the compounds in the sample were calculated using Eq. (2):

2 $${C}_{s}={C}_{t}\times \frac{{x}_{s}}{{x}_{t}}\times \frac{{m}_{t}}{{m}_{s}}\times \frac{{w}_{s}}{{w}_{t}}$$

where *C*_*s*_ is the concentration of the analyte in the sample (unknown); *C*_*t*_ is the known concentrations of the tracer; *m*_*s*_ and *m*_*t*_ are the masses taken from the sample and the tracer during sample preparation, and *w*_*s*_ and *w*_*t*_ are the molecular weights of the natural and labelled analytes, respectively.

## Results and discussion

The set-up of an IDMS procedure employing Eqs. (1) and (2) requires the knowledge of the isotope composition of the natural and labelled analytes. Moreover, the capabilities of the mass spectrometer to accurately measure isotope distributions must be assessed in both selected ion monitoring (SIM) and multiple reaction monitoring (MRM) modes. Typical errors in quadrupole-based instruments are tailing at the low mass side (in ESI–LC–MS approaches) and lack of purity of the measured cluster (in EI–GC–MS approaches) [15]. Otherwise, isotope measurements in quadrupole-based instruments are remarkably accurate [17].

### Measurement of the fragmentation pattern and isotopic enrichment of amino acids by LC–ESI–MS/MS and GC–EI–MS/MS

Amino acids as butyl ester derivatives were separated using the optimized chromatographic conditions presented in Table S1 of the Supporting Information. To study the spectral purity of the natural abundance compounds and isotopic enrichment of their labelled counterparts, the whole cluster of the molecular ion was measured in SIM mode by injecting 1 µg g^−1^ mixtures of amino acids into the LC–MS/MS system. Table 1 presents the results for the amino acids ordered by increasing retention time. The measured cluster was found to be pure (molar fractions ${\upchi }_{{\text{M}}}$ ranging from 99.55% to 100.01%), with a minor tailing at the low mass side of the spectrum (${\upchi }_{{\text{M}}-1})$ accounting for 0.17 to 0.41% of the isotopic signature measured. Based on those data, along with the measured isotope distribution of the labelled compounds, their isotopic enrichments were calculated [15] and are also given in Table 1.

**Table 1:** Retention time (RT), measured cluster ion (SIM), fragment formula, spectral purity (uncertainty as standard deviation of five injections) and isotopic enrichment determined by LC–ESI–MS/MS for the reported amino acids. The uncertainty values of the molar fractions X_M_ and X_M-1_ and the isotopic enrichments were calculated as the combined uncertainties of five intra-day injections in the LC–ESI–MS/MS system

Compound	RT (min)	[M + H] (m/z)	Cluster (m/z)	Protonated molecular formula	${\upchi }_{{\text{M}}-1}$ ± s (%)	${\upchi }_{{\text{M}}}$ ± s (%)	Fragment formula for the labelled compound	Isotopic enrichment $\pm$ u (%)
Arginine	0.7	231	230–235	C_10_H_22_N_4_O_2_	0.28 ± 0.10	99.69 ± 0.10	C_10_H_22_^15^N_4_O_2_	98.9 ± 0.1
Proline	2.6	172	171–176	C_9_H_17_NO_2_	0.41 ± 0.05	99.55 ± 0.07	^13^C_1_^12^C_8_H_17_NO_2_	99.9 ± 0.1
Valine	4.0	174	173–178	C_9_H_17_NO_2_	0.27 ± 0.10	99.91 ± 0.10	^13^C_1_^12^C_8_H_17_NO_2_	99.9 ± 0.1
Tyrosine	4.5	238	237–242	C_13_H_19_NO_3_	0.30 ± 0.07	99.86 ± 0.09	^13^C_2_^12^C_11_H_19_NO_3_	98.9 ± 0.1
Leucine	5.0	188	187–192	C_10_H_21_NO_2_	0.31 ± 0.09	99.65 ± 0.20	^13^C_1_^12^C_9_H_21_NO_2_	99.9 ± 0.4
Isoleucine	5.0	188	187–192	C_10_H_21_NO_2_	0.31 ± 0.14	99.67 ± 0.19	^13^C_1_^12^C_9_H_21_NO_2_	99.0 ± 0.2
Phenylalanine	5.6	222	221–226	C_13_H_19_NO_2_	0.17 ± 0.10	100.01 ± 0.14	^13^C_1_^12^C_12_H_19_NO_2_	99.4 ± 0.1

On the other hand, amino acids as TBDMS derivatives were separated by gas chromatography using the optimized chromatographic conditions presented in Table S2 of the Supporting Information. It was reported by several authors that arginine TBDMS derivative is unstable [18–20], but its isotopic composition could be measured here. In this case, the cluster of the molecular ion could not be measured, as the loss of a ter-butyl group in the source was typical for all amino acids except arginine, where a more extensive fragmentation was observed with the loss of two nitrogen atoms. Thus, the most abundant cluster was measured in SIM mode by injecting 1 µg g^−1^ mixtures of amino acids into the GC–MS system. Table 2 presents the data obtained ordered by increasing retention time of the amino acids. The most remarkable fact is that the measured cluster is clearly not pure for the amino acids, with a significant component of the protonated molecular ion [M + H]^+^ together with the molecular ion [M]^+^. The contribution of [M + H] ranged from 1.07% for tyrosine to 2.84% for isoleucine. Nevertheless, by considering their fragmentation pattern [15], their isotopic enrichment could be calculated and is also given in Table 2. As can be observed, the isotopic enrichments measured agree with those values obtained by LC–ESI–MS/MS.

**Table 2:** Retention time (RT), measured cluster ion (SIM), fragment formula, spectral purity (uncertainty as standard deviation of five injections) and isotopic enrichment determined by GC–MS for the reported amino acids. The uncertainty values of the molar fractions X_M_ and X_M+H_ and the isotopic enrichments were calculated as the combined uncertainties of five intra-day injections in the GC–MS system

Compound	RT (min)	[M] (m/z)	Cluster (m/z)	Fragment formula	${\upchi }_{{\text{M}}}$ ± s (%)	${\upchi }_{{\text{M}}+{\text{H}}}$ ± s (%)	Fragment formula for the labelled compound	Isotopic enrichment $\pm$ u (%)
Arginine	6.36	213	211–218	C_9_H_21_N_2_Si_2_	96.77 ± 0.48	2.49 ± 0.48	C_9_H_21_^15^N_2_Si_2_	98.7 ± 0.2
Valine	8.10	288	286–291	C_13_H_30_NO_2_Si_2_	97.88 ± 0.28	1.75 ± 0.28	^13^C_1_^12^C_12_H_30_NO_2_Si_2_	100.0 ± 0.3
Leucine	8.37	302	300–305	C_14_H_32_NO_2_Si_2_	97.19 ± 0.33	2.53 ± 0.33	^13^C_1_^12^C_13_H_32_NO_2_Si_2_	99.9 ± 0.3
Isoleucine	8.59	302	300–305	C_14_H_32_NO_2_Si_2_	96.88 ± 0.39	2.84 ± 0.39	^13^C_1_^12^C_13_H_32_NO_2_Si_2_	98.9 ± 0.4
Proline	8.95	286	284–289	C_13_H_28_NO_2_Si_2_	98.34 ± 0.30	1.3 ± 0.28	^13^C_1_^12^C_12_H_28_NO_2_Si_2_	100.0 ± 0.3
Phenylalanine	11.15	336	334–339	C_17_H_30_NO_2_Si_2_	98.30 ± 0.26	1.52 ± 0.25	^13^C_1_^12^C_16_H_30_NO_2_Si_2_	99.4 ± 0.3
Tyrosine	14.0	466	464–470	C_23_H_44_NO_3_Si_3_	98.51 ± 0.35	1.07 ± 0.35	^13^C_2_^12^C_21_H_44_NO_3_Si_3_	98.5 ± 0.4

### Selection of MRM transitions by LC–ESI–MS/MS and GC–EI–MS/MS

Figure S4 shows the LC and GC chromatograms for the separation of amino acid mixtures at 10 µg g^−1^ detected in MRM mode. In LC–ESI–MS/MS, the fragmentation of the amino acids was studied to select those MRM transitions providing the best sensitivity and specificity. The protonated molecular ion [M + H]^+^ was selected as precursor ion for all amino acids. Product ions [M-56]^+^, arising from the loss of C_4_H_8_ by collision induced dissociation (CID), were selected for valine, proline, tyrosine, leucine and phenylalanine due to their high intensity, whereas the product ion [(M + H)-17]^+^ (loss of NH_3_) was selected for arginine. Due to co-elution of leucine and isoleucine, for mixtures containing both amino acids, i.e. angiotensin I hydrolysates, a specific and unique product ion [(M + H)-119]^+^ (loss of the main chain) was selected for the specific measurement of isoleucine.

To evaluate the capabilities of the triple quadrupole instrument to accurately measure MRM transitions, theoretical isotope distributions of in-cell fragment ions were calculated using suitable MRM-dedicated software such as IsoPatrn^®^ [21] and the results compared with the experimental values. Table S4 in Supporting Information shows that for both natural and labelled amino acids, there is good agreement between the experimental and theoretical isotopic composition. The uncertainty in experimental values is indicated as the standard deviation of five independent LC–MS/MS injections. Finally, the experimental abundance values were employed for further IDMS calculations.

In the same way, the fragmentation of the amino acids was studied by GC–MS/MS to select those MRM transitions providing the best sensitivity and specificity. The in-source-generated radical cation [M-C(CH_3_)_3_]^+**·**^ was selected as precursor ion for all amino acids, whereas product ions [M-CO–C(CH_3_)_3_]^+**·**^ were selected for valine, proline, tyrosine, leucine, isoleucine and phenylalanine due to their high intensity. For arginine, the product ion selected was the loss of CH_2_N_2_, [M-CH_2_N_2_-C(CH_3_)_3_]^+**·**^. It is remarkable that for GC–MS/MS, most product ions lose their labelled atom, as the ^13^C label is usually present in the carboxylic group. Theoretical isotope distributions of in-cell fragment ions were also calculated with IsoPatrn^®^ and compared with the experimental values. Table S5 in the Supporting Information shows that for both natural abundance and labelled amino acids, there is good agreement between the experimental and theoretical isotopic composition. The uncertainty in experimental values is indicated as the standard deviation of five independent GC–MS/MS injections. Finally, experimental abundance values were employed for further IDMS calculations.

### Optimization of the peptide hydrolysis method

First, labelled amino acids were quantified by reverse isotope dilution, using the CRM natural abundance amino acid mix solution (solution of 17 amino acids). Then, two reference materials, angiotensin I and C-peptide, were spiked with the labelled amino acids and subjected to different hydrolysis conditions. Angiotensin I is a decapeptide with the amino acid sequence DRVYIHPFHL, whereas C-peptide is a 31-mer peptide with the amino acid sequence EAEDLQVGQVELGGGPGAGSLQPLALEGSLQ.

In order to quantify angiotensin I and C-peptide hydrolysates by LC–MS/MS, two different mixtures of labelled amino acids were required because of co-elution of leucine and isoleucine in LC determinations. As described previously, isoleucine can be measured in the presence of leucine due to a specific MRM transition. However, to measure leucine in C-peptide hydrolysates, a mixture of labelled amino acids without isoleucine was required. On the other hand, for GC–MS/MS determinations, a unique mixture of all amino acids was employed, as there was no co-elution of amino acids in the GC separation. Unfortunately, arginine, isoleucine and tyrosine are not present in the amino acid sequence of C-peptide and could not be employed for quantification.

Purity assessment of proteins and peptides has been classically performed through acid hydrolysis using sealed T-hydrolysis tubes, a strong acid (mainly 6 M HCl) and long incubation times (24–72 h) [7, 22–28]. Therefore, we first tried to evaluate this well-known and established hydrolysis method using angiotensin I SRM 998. Different hydrolysis times (24, 48, 60 and 78 h) were studied and the content of the amino acids in the different hydrolysates were quantified by GC–MS/MS. The obtained recovery results are presented in Figure S5 of the Supporting Information. As can be seen, a minimum of 48 h of hydrolysis was required to obtain complete cleavage in the measured amino acids.

**Fig. 1:**
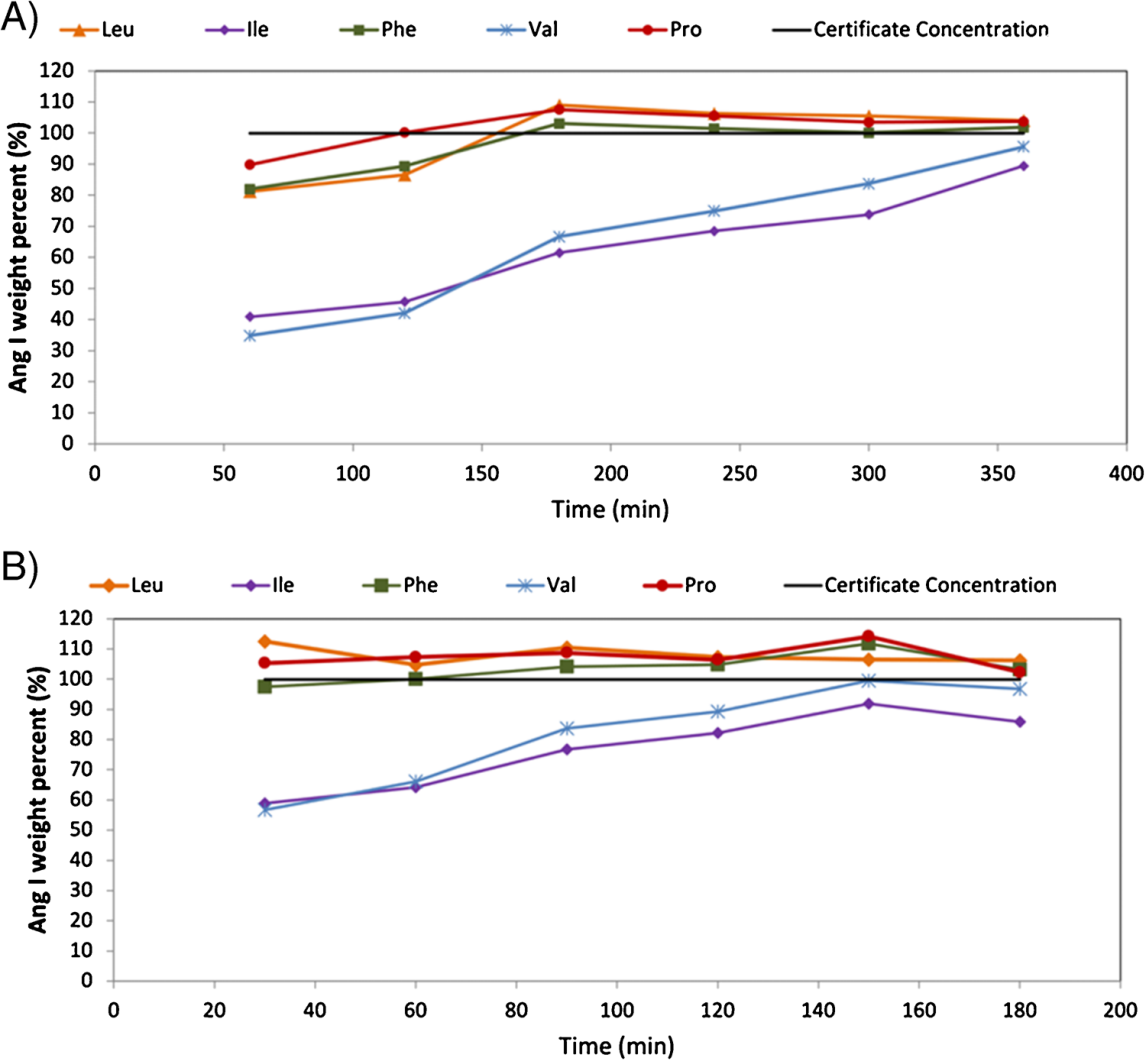
Focused microwave-assisted hydrolysis efficiency obtained for amino acids Leu, Ile, Phe, Val and Pro at different times from 30 to 360 min and temperatures of (**A**) 130 °C and (**B**) 150 °C. Amino acid quantification was performed by GC–MS/MS

Recently, various authors have made use of focused microwaves to decrease the hydrolysis time of proteins and peptides in comparison to the classical time-consuming procedures [29–32]. In our experiments, two parameters were optimized: the hydrolysis temperature and the hydrolysis time. First, we performed the hydrolysis of a solution of 100 ± 4 µg g^−1^ SRM at 130 °C for 60, 120, 180, 240, 300 and 360 min, and calculated for each time the hydrolysis percentage according to theoretical estimated amounts for free amino acids. Then, the same experiment was performed for a temperature of 150 °C, but using shorter hydrolysis times: 30, 60, 120, 150 and 180 min. The results for these two experiments are presented in Fig. 1 using amino acids leucine, isoleucine, phenylalanine, valine and proline and GC–MS/MS determination of the cleaved amino acids. As can be observed, leucine, proline and phenylalanine are fully hydrolysed even with short irradiation times, while valine and isoleucine required a long hydrolysis time of 360 min to be liberated at 130 °C (Fig. 1A). However, they are fully hydrolysed after 150 min at 150 °C (Fig. 1B). This pronounced decrease in hydrolysis time (from 48 h to ca. 2.5 h) has been reported by other authors [7] based not only on the temperature used but also on other factors such as the identity of amino acids, the structure of the peptide or the acid used.

**Fig. 2:**
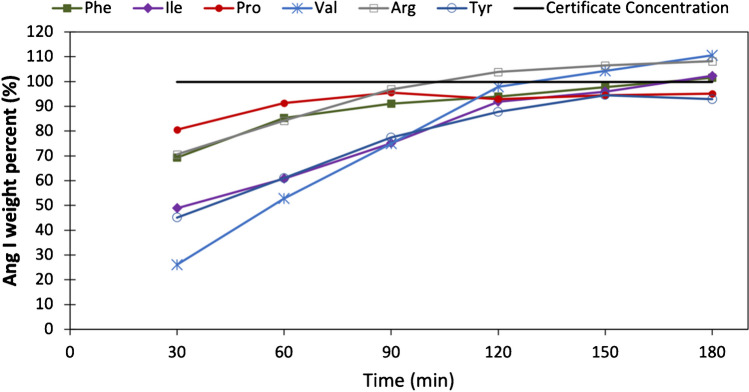
Focused microwave-assisted hydrolysis efficiency obtained for amino acids Arg, Ile, Phe, Tyr, Val and Pro at different times from 30 to 180 min at 150 °C. Amino acid quantification was performed by LC–MS/MS

The hydrolysis was repeated at 150 °C using now LC–MS/MS determinations for arginine, isoleucine, phenylalanine, tyrosine, valine and proline. The results obtained are presented in Fig. 2. As can be seen, the hydrolysis curves for all amino acids reached a plateau of quantitative hydrolysis after 150 min, in agreement with Fig. 1B. In general, the results of the measurements by GC–MS/MS and LC–MS/MS are comparable, but a larger number of amino acids can be measured by LC–MS/MS. These results are also similar to those obtained by other authors for the same amino acids and under similar hydrolysis conditions [11, 33, 34]. Considering the results obtained, it was decided to establish a time of 150 min for the hydrolysis time and 150 °C as the hydrolysis temperature.

**Fig. 3:**
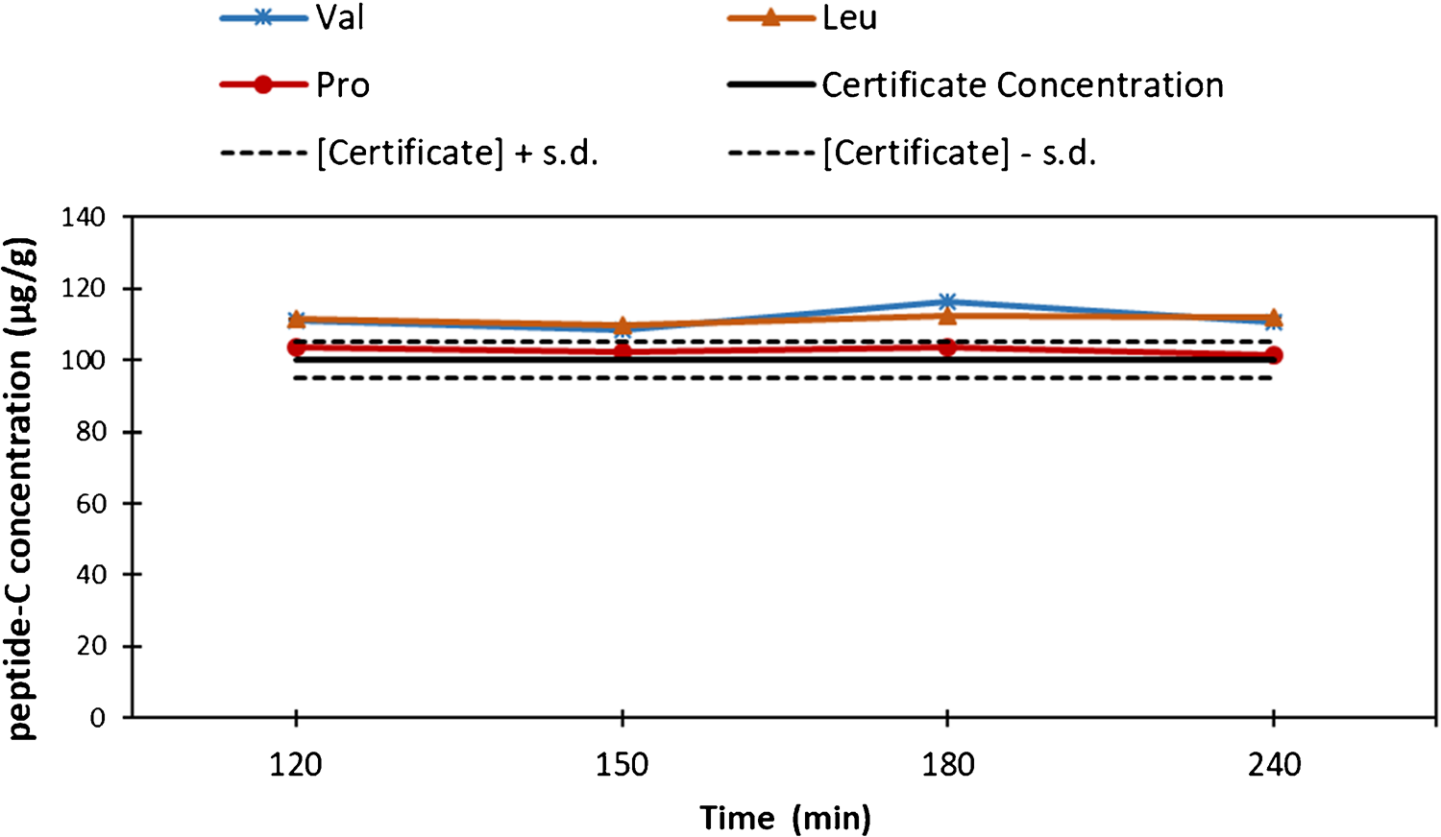
Efficacy of microwave-assisted hydrolysis of C-peptide obtained at different times (from 90 to 240 min) at 150 °C for amino acids V, L and P. Amino acid quantification was performed by isotope dilution LC–MS/MS

We also studied the hydrolysis behaviour of the C-peptide (CRM 6901-c NMIJ). C-Peptide is a longer peptide, containing 31 amino acids, and therefore we expected a longer hydrolysis time. We assayed hydrolysis times of 90, 120, 150, 180 and 240 min. Hydrolysates were only measured by LC–MS/MS due to the scarcity of the material, and only three amino acids were measured, as arginine, isoleucine and tyrosine are not present in the amino acid sequence of C-peptide. The results obtained are presented in Fig. 3. It can be seen that at 120 min, proline, valine and leucine were already cleaved and reached a constant concentration. These results agree with those found by other authors [28]. Interestingly, these amino acids present the same behaviour in angiotensin I. According to the obtained results, we confirm that complete cleavage for the measured amino acids in angiotensin I and C-peptide would be achieved in 150 min using 150 °C as the optimal conditions.

### Reproducibility and accuracy of the focalized microwave hydrolysis method

Once the hydrolysis conditions were optimized, five hydrolysis were performed for CRM angiotensin I (100 ± 4 µg g^−1^) to evaluate the reproducibility of the method. Five injections of the same hydrolysate (repeatability) and from different hydrolysates (intra-day and inter-day reproducibility) were performed to evaluate the whole methodology. Additionally, blank values (*n* = 5) were used to further calculate the limits of detection (LOD) of the method for each amino acid. These experiments were performed in duplicate, and one set of samples was measured by LC–MS/MS whereas the other set was measured by GC–MS/MS. The obtained reproducibility results (intra-day) are presented in Table 3 for LC–MS/MS and for GC–MS/MS.

**Table 3:** Concentrations obtained for a 100 ± 4 µg g^−1^ angiotensin I solution using the six amino acids measured by LC–MS/MS and GC–MS at 150 °C and 150 min hydrolysis. Five injections of different hydrolysates (intra-day reproducibility) were performed

LC-MS/MS							
Hydrolysis	Angiotensin I concentration ± s (µg g^−1^)						
	Phenylalanine	Isoleucine	Proline	Valine	Tyrosine		Arginine
1	101.2 ± 1.3	90.6 ± 0.7	95.4 ± 0.4	99.4 ± 3.8	91.2 ± 1.3		104.7 ± 2.6
2	112.3 ± 1.0	98.9 ± 0.8	99.3 ± 1.0	104.7 ± 3.8	100.5 ± 1.3		110.5 ± 4.2
3	101.2 ± 1.2	92.0 ± 5.0	93.7 ± 0.3	93.0 ± 1.1	91.7 ± 0.6		102.8 ± 0.8
4	100.4 ± 1.4	97.3 ± 0.3	93.4 ± 0.2	102.2 ± 0.7	86.7 ± 2.5		108.3 ± 1.3
5	100.7 ± 0.3	99.0 ± 1.2	94.1 ± 0.6	102.6 ± 1.2	86.8 ± 1.1		109.2 ± 0.9
Average	**103.2 ± 5.1**	**95.6 ± 4.0**	**95.2 ± 2.4**	**100.4 ± 4.5**	**91.4 ± 5.6**		**107.1 ± 3.2**
LOD	**0.27**	**0.15**	**0.13**	**0.16**	**0.31**		**0.18**
GC–MS							
Hydrolysis	Angiotensin I concentration ± s (µg g^−1^)						
	Phenylalanine	Isoleucine	Proline	Valine		Leucine	
1	121.9 ± 15.7	93.2 ± 3.0	95.8 ± 1.2	97.5 ± 0.6		110.1 ± 5.2	
2	106.6 ± 6.3	91.8 ± 2.0	96.8 ± 3.0	94.0 ± 1.1		110.6 ± 0.9	
3	98.9 ± 1.7	92.1 ± 1.0	93.8 ± 1.9	94.4 ± 1.4		117.1 ± 8.2	
4	107.3 ± 1.5	93.9 ± 1.8	94.4 ± 0.3	95.8 ± 0.6		113.2 ± 4.8	
5	105.7 ± 11.8	100.2 ± 2.5	112.3 ± 1.6	110.7 ± 2.1		130.9 ± 4.8	
Average	**108.0 ± 8.4**	**94.2 ± 3.5**	**98.6 ± 7.8**	**98.5 ± 7.0**		**116.4 ± 8.6**	
LOD	**0.80**	**0.43**	**0.20**	**0.19**		**0.43**	

As the concentration of angiotensin I in the test solution was 100 ± 4 µg g^−1^, it is also possible to numerically evaluate the recovery for each amino acid. Thus, in LC determinations (Table 3), phenylalanine, valine, proline and isoleucine presented good recoveries, but arginine values were slightly higher (107.1 ± 3.2 μg g^−1^) and tyrosine values slightly lower (91.4 ± 5.6 µg g^−1^) than the certified value. The relative standard deviation (RSD) between injections was around 1% for most amino acids, while hydrolysis reproducibility was between 2.5 and 6.1%, depending on the amino acid selected.

On the other hand, for the GC determinations, proline and valine provided good recovery values, while leucine (116.4 ± 8.6 µg g^−1^) and phenylalanine (108.0 ± 8.4 µg g^−1^) values were above the expected range and isoleucine values (94.2 ± 3.5 µg g^−1^) were lower than expected. In general, GC–MS/MS determinations provided RSD values higher than those provided by LC–MS/MS in terms of both repeatability of injections and intra-day reproducibility.

In summary, LC–MS/MS data seemed to be of higher quality than those obtained by GC–MS/MS, also providing lower limits of detection for the selected amino acids. Finally, the inter-day variability for angiotensin I hydrolysis and amino acid determination was performed only by LC–MS/MS. For this experiment, five sample replicates were hydrolysed on each of three different days. The results obtained are presented in Table 4. Again, intra-day repeatability, measured as RSD, was between 1 and 5% for all amino acids. Furthermore, inter-day reproducibility was better than 5% for all amino acids, which can be considered satisfactory. Overall, the amino acid valine provided the best values in terms of recovery for the data shown in Tables 3 and 4, but other amino acids also showed promising results.

**Table 4:** Concentrations obtained for a 100 ± 4 µg g^−1^ angiotensin I solution using the six amino acids measured by LC–MS/MS at 150 °C and 150 min hydrolysis. Inter-day reproducibility

	Angiotensin I concentration ± s (µg g^−1^)					
	Phenylalanine	Isoleucine	Proline	Valine	Tyrosine	Arginine
Day 1	103.2 ± 5.1	95.6 ± 4.0	95.2 ± 2.4	100.4 ± 4.5	91.4 ± 5.6	107.1 ± 3.2
Day 2	104.3 ± 1.1	90.4 ± 3.8	93.4 ± 0.9	97.9 ± 3.2	85.7 ± 4.6	100.2 ± 2.9
Day 3	98.0 ± 4.5	91.2 ± 2.6	93.5 ± 1.3	98.5 ± 2.5	85.7 ± 3.2	99.3 ± 1.5
Average	101.8 ± 3.4	92.4 ± 2.8	94.0 ± 1.0	98.9 ± 1.3	87.6 ± 3.3	102.2 ± 4.3

### Synthesis, purification, and characterization of naturally abundant and ^13^C_1_-labelled angiotensin I

Isotopically labelled peptides containing only one or two ^13^C atoms were employed previously in our laboratory for the determination of cystatin C in blood serum [35] and urine [36] and for the determination of different proteins in human serum [37]. These minimally labelled peptides (mass overlapping peptides, MOPs) have the advantage that both the natural peptide and labelled analogue have the same retention time in LC separations and can be transmitted through the first quadrupole using a relatively narrow transmission window, particularly for multiply charged ions, allowing the simultaneous fragmentation of both the natural abundance and the labelled analogue in the collision cell. Also, these minimally labelled peptides are perfectly suited for the application of Eqs. (1) and (2) in IDMS.

**Fig. 4:**
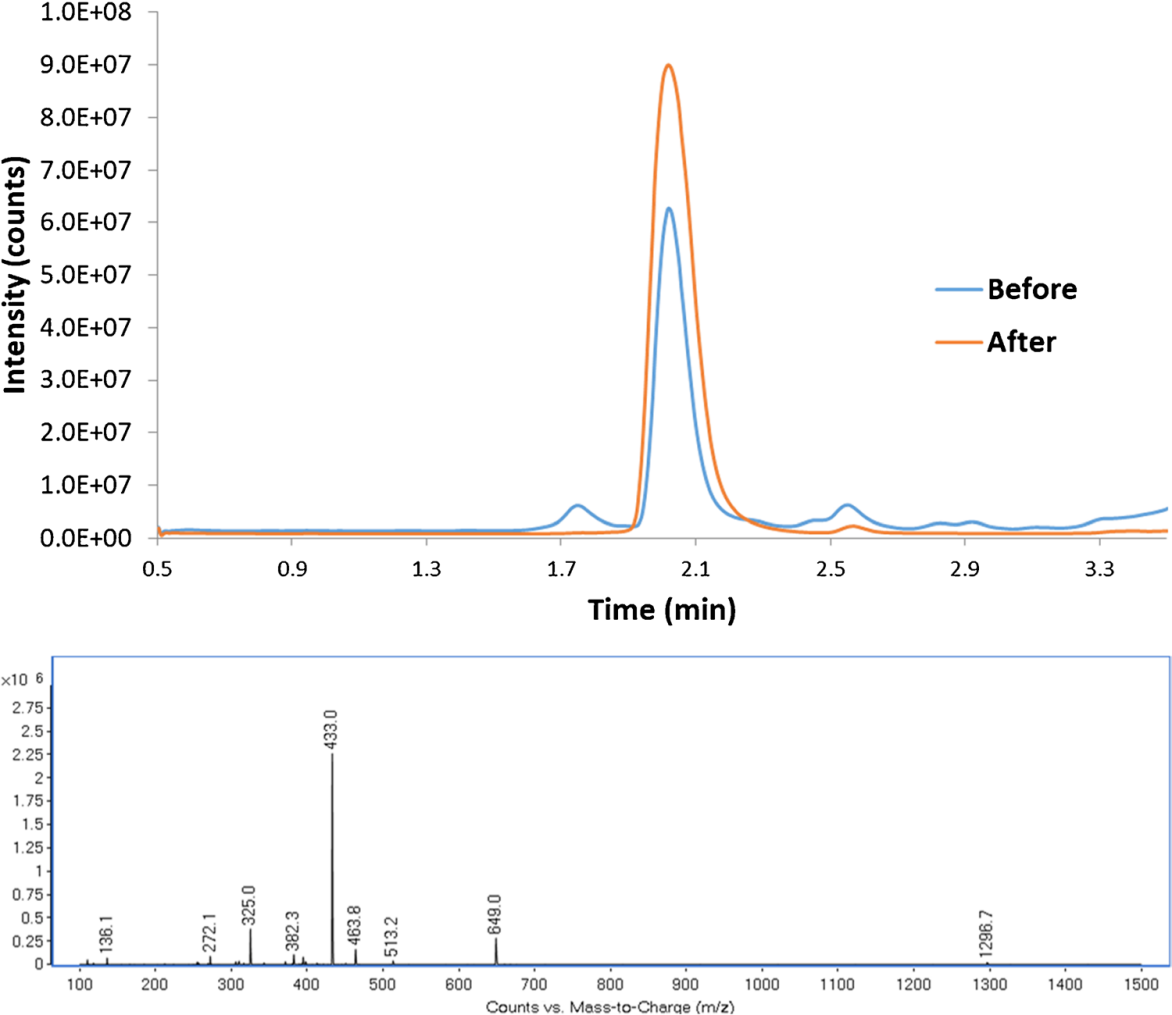
Chromatogram of the synthesized natural abundance angiotensin I before and after purification by semi-preparative LC (top) and the mass spectrum obtained at the retention time of the more intense chromatographic peak (bottom). The main peak corresponds to the triply charged [M + 3H] ion at 433.0 m/z

Therefore, we performed the synthesis of natural and labelled angiotensin I (using ^13^C_1_-L-valine) and the SPPS procedure [14] described. The synthesis products were characterized using the same LC conditions employed for the separation of amino acids. Figure 4 presents the overlapped chromatograms of natural angiotensin before and after purification by preparative LC (top) and the scan mass spectrum (range 100–1300) of the purified synthesis product (bottom). As can be seen, the theoretical mass-to-charge 1297 appears in the natural product as expected. Furthermore, ions providing the highest signal for naturally abundant angiotensin I were 649.0 and 433.0, corresponding to the double-charged [M + 2H]^2+^ and triple-charged [M + 3H]^3+^ molecular ions, respectively, whereas for labelled angiotensin I these ions were shifted 0.5 and 0.3 m/z units, respectively.

### Measurement of the isotope composition of natural and labelled angiotensin I by LC–ESI–MS/MS and selection of MRM conditions

The spectral purity and isotope composition of naturally abundant synthetic angiotensin I was measured in both SIM and MRM modes. In SIM mode, the molecular ion was measured in the cluster at 1295–1300 m/z ([M + H]^+^]). Five replicates of 100 µg g^−1^ were injected (1 µL) into the LC–MS/MS system. The retention time for angiotensin I was 2.1 min. Table 5 presents the results for the spectral purity of the natural molecular ion and the resulting isotope enrichment for the labelled angiotensin I. It must be noted that a large tailing at the low mass side of the spectrum was obtained (1.51%), which is to be expected at the mass range measured (ca. 1300 m/z) in quadrupole instruments. Considering this tailing, the resulting isotope enrichment for ^13^C in the labelled angiotensin I was 99.2 ± 0.1%.

**Table 5:** Spectral purity obtained for the synthesized natural angiotensin I in both SIM and MS/MS modes. The isotopic enrichment obtained for the synthesized isotopically enriched angiotensin I by the different acquisition modes is also given. The uncertainty of the results corresponds to the standard deviation of five injections in the LC–ESI–MS/MS system (cluster purity) or total combined uncertainty (isotope enrichment)

Acquisition mode	Protonated molecule formula	Cluster (m/z)	X_M_ ± s (%)	X_M-1_ ± s (%)	Isotopic enrichment ± u (%)
SIM	C_62_H_90_N_17_O_14_	1295–1302	98.61 ± 0.16	1.51 ± 0.12	99.2 ± 0.2
MRM	C_29_H_45_N_7_O_8_	619–624	99.49 ± 0.11	0.48 ± 0.02	99.1 ± 0.1

For MRM, the triple-charged ion at m/z 433.0 was selected as precursor ion due to its high intensity. Figure S6 in the supporting information shows the mass spectra obtained when fragmenting both natural (top) and labelled (bottom) angiotensin I. As can be observed, the fragment ions b_4_^+^ ([DRVY]-NH_3_, m/z 534.3), a_5_^+^ ([DRVYI]-CO-NH_3_ m/z 619.3) and b_5_^+^ ([DRVYI]-NH_3_, m/z 647.3) provide high intensities and were selected to perform further isotopic measurements. Furthermore, as these fragments contain ^13^C_1_-valine (labelling amino acid), labelled angiotensin I product ions will present a mass shift of one mass unit (see Figure S6). The selected MRM transitions were measured using low resolution in the first quadrupole to transmit the whole cluster to the collision cell, as described elsewhere [15] and shown in Figure S7 for cluster 647. Unfortunately, the fragment ion b_4_^+^ ([DRVY]-NH_3_, m/z 534.3) was affected by a minor spectral interference at M-2 and could not be employed to calculate the isotope enrichment. The data on the spectral purity of the natural fragment ion a_5_^+^ ([DRVYI]-CO-NH_3_ m/z 619.3) and the resulting isotope enrichment for the labelled angiotensin I are also presented in Table 5. A small contribution of tailing at the low mass side is still observed in MRM. It can be seen that isotope enrichment results by MRM agree well with those obtained by SIM.

For the transmission of the whole ion cluster to the collision cell and the accurate measurement of the isotopic composition of mixtures of natural and labelled angiotensin I in product ions, the mass resolution of the first quadrupole was modified by adjusting the gain and the width offset. The optimization process is shown in Figure S7 of the Supporting Information. The basic idea is to select a mass-to-charge window in the first quadrupole that transmits the whole ion cluster to the collision cell, as described previously [34]. Considering these results, we decided to use a gain offset of 10 and a width offset of 2 in the first quadrupole to perform the following measurements, as these values provide an intermediate value for window width (7 units), which we consider sufficient to transmit the whole cluster, for both natural and labelled compounds. Here it is important to note that the wider the window, the larger the number of possible transmitted interferences.

Finally, the theoretical isotope distributions of the selected in-cell fragment ions were compared with those obtained experimentally by several injections of individual solutions of natural and labelled peptides for both transitions. The resolution in the third quadrupole was kept at its nominal “unit” value. The results are presented in Table S6 of the Supporting Information. As can be seen, there is good agreement between the theoretical and experimental values, demonstrating the accurate isotopic measurement capabilities of the developed methodology.

### Quantification of labelled angiotensin I in its native form by IDMS

The SRM 998 angiotensin I solution was used to determine the concentration of a gravimetrically weighed solution of the synthetic—and purified—labelled angiotensin I by reverse IDMS. The three clusters and four transitions shown in Table S6 were employed for quantification using Eqs. (1) and (2). The experimental abundance values shown in Table S6 for natural and labelled angiotensin I were employed in Eq. (1). A concentration of 60 ± 2 µg g^−1^ of labelled synthetic angiotensin I was found.

### Comparison of IDMS by amino acid analysis and in native form

**Fig. 5:**
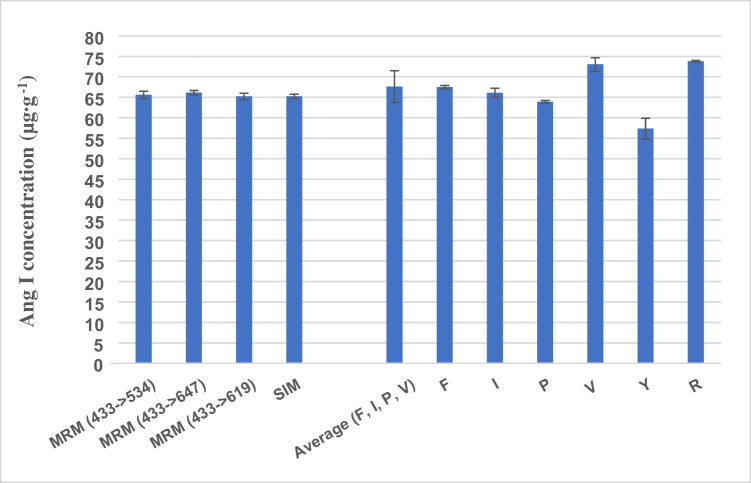
Concentrations of synthesized natural angiotensin I obtained by LC-MS/MS using three different MRM transitions, by LC-MS/MS in the SIM mode and by GC–MS using different amino acids as labelled analogues and the average obtained from the individual GC–MS determinations

The developed methods were applied to assess the concentration of a solution containing ca. 70 µg g^−1^ of natural synthetic angiotensin I. First, this solution was hydrolysed using the optimized conditions and its concentration was determined via AAA using the developed IDMS LC–MS/MS method. Second, the labelled angiotensin I was spiked to the natural form and the natural synthetic angiotensin I quantified by LC–MS/MS in its native form using both MRM and SIM. The results for the obtained concentrations using the described methods are presented in Fig. 5.

As can be seen, the results obtained by the two procedures mostly coincide. The results obtained for the different amino acids are consistent with those found during the optimization of the hydrolysis. If we assume that the data based on the native IDMS are correct, then the peptide concentrations found from the analysis of tyrosine and arginine in the hydrolysates are 90 and 110% of the expected value, respectively. A higher-than-expected value was also found using the valine concentration in the hydrolysates to calculate the angiotensin I concentration. In general, most publications do not consider tyrosine and arginine to be stable and reliable amino acids for peptide quantification [7, 38]. Thus, the average of the remaining four amino acids was chosen, obtaining a value that agrees well with that obtained following the native IDMS strategy.

As described by Stocks et. al., the values of the different amino acids can be combined (averaged) to obtain a single purity value (concentration) of a peptide [39]. However, there is still no consensus on the most appropriate statistical model to obtain this value, since the number of amino acids used in the determination and their identity is decisive in the final result. Some authors only consider it appropriate to use the values obtained for “stable” amino acids, such as proline and phenylalanine [11, 28, 40]. However, this criterion is vague, since there is also no consensus on which amino acids are more stable. The stability of the amino acids will depend on the hydrolysis conditions: time, temperature, pH, concentration of the hydrolysis agent, etc. In our case, the most suitable amino acids to obtain the most accurate average value of the purity of angiotensin I were phenylalanine, valine, isoleucine and proline. Likewise, these amino acids also provided accurate and reproducible values in C-peptide hydrolysis (in this case, leucine was measured instead of isoleucine).

However, obtaining a peptide purity value through the average of n individual amino acids results in high uncertainty, since the result depends on n independent determinations, each with its own uncertainty. Therefore, averaging individual amino acid values is not recommended if highly accurate results are required. Likewise, the uncertainty values are similar to those published by other authors [20, 23, 41].

## Conclusions

In this work we optimized a hydrolysis procedure using microwaves for the acid hydrolysis of two different peptides: angiotensin I and C-peptide. Hydrolysates were quantified by HPLC–MS/MS and GC–MS/MS methods and AAA using a CRM and a SI-traceable approach through isotope dilution. As reported by other authors, leucine, proline and phenylalanine were quickly cleaved from the studied peptides, whereas valine and isoleucine required at least 150 min at 150 °C using microwaves to be 100% cleaved. On the other hand, tyrosine reached a plateau at 90% of the expected value, whereas arginine was 10% over the expected value. The developed focused microwave method decreased the hydrolysis time from 48 h for the classical procedure to 150 min.

Natural and labelled angiotensin I were successfully synthesized and purified using semi-preparative HPLC, and an MS/MS method using low resolution in the first quadrupole was developed for the measurement of angiotensin I through isotope dilution. This method permits the transmission of the whole cluster at the same time for both natural and labelled peptides, which is indispensable to ensure accurate measurements for non-resolved MS peaks.

The developed methods were used to quantify the synthetic angiotensin I through two different approaches. The results obtained provided values that were in agreement, which validates the methodology and opens the way for use of the synthesized angiotensin I peptide as an “in-house” traceable standard to validate future AAA procedures for the determination of the purity of other peptides of interest. We analysed the two certified reference materials commercially available, with satisfactory results. At this point, no further validation of the methodology can be carried out, and thus we must assume that the hydrolysis conditions optimized in this work can be safely applied to other peptide standards. Also, the naturally abundant synthesized peptide can be employed as a quality control when determining the purity of other peptide standards.

### Supplementary Information

Below is the link to the electronic supplementary material.

Supplementary file1 (DOCX 506 KB)
