# Supplementary material for: Evaluation of different isotope dilution mass spectrometry strategies for the characterization of naturally abundant and isotopically labelled peptide standards
Source: Anal Bioanal Chem. 2024 Feb 16;416(7):1717–31. doi: 10.1007/s00216-024-05176-1 (PMC10899365; doi:10.1007/s00216-024-05176-1)

**ELECTRONIC SUPPORTING INFORMATION**

**Evaluation of different isotope dilution mass spectrometry strategies for the characterisation of naturally abundant and isotopically labelled peptide standards**

Jesús Nicolás Carcelén^1^, Helí Potes Rodríguez^1^, Adriana González-Gago^1^, Juan Manuel Marchante-Gayón*^1^, Alfredo Ballesteros^2^, José Manuel González^2^, José Ignacio García Alonso^1^ and Pablo Rodríguez-González^1^

*^1^Department of Physical and Analytical Chemistry. Faculty of Chemistry. University of Oviedo. Spain.*

*^2^Department of Organic and Inorganic Chemistry. Faculty of Chemistry. University of Oviedo. Spain.*

*To whom correspondence should be addressed: [marchant@uniovi.es](mailto:marchant@uniovi.es)

Number of Pages: 15

Number of Figures: 7

Number of Tables: 6

**
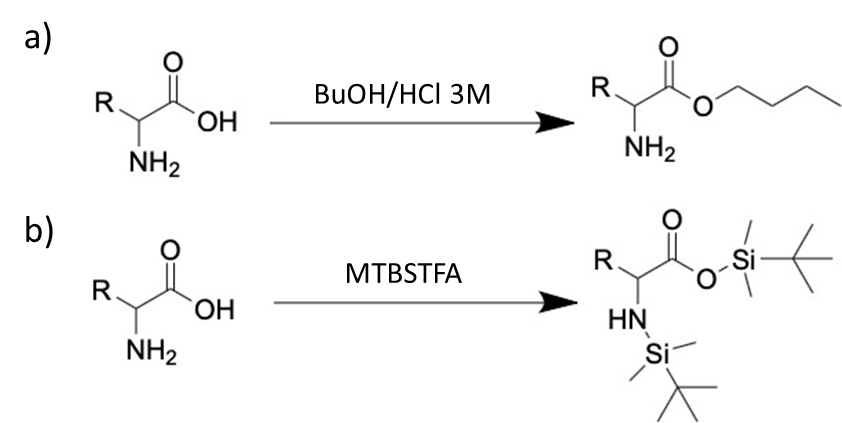
**

**Figure S1**.- Amino acids derivatization reactions: a) esterification for their determination by LC-MS/MS and b) silanization for their determination by GC-MS/MS.


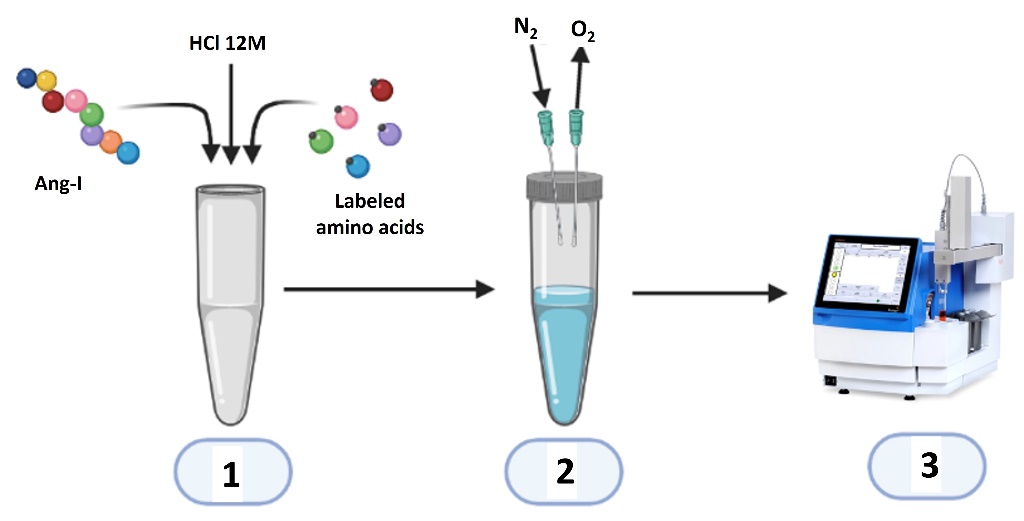


**Figure S2**.- Sample preparation procedure for the focused microwave assisted peptide hydrolysis.


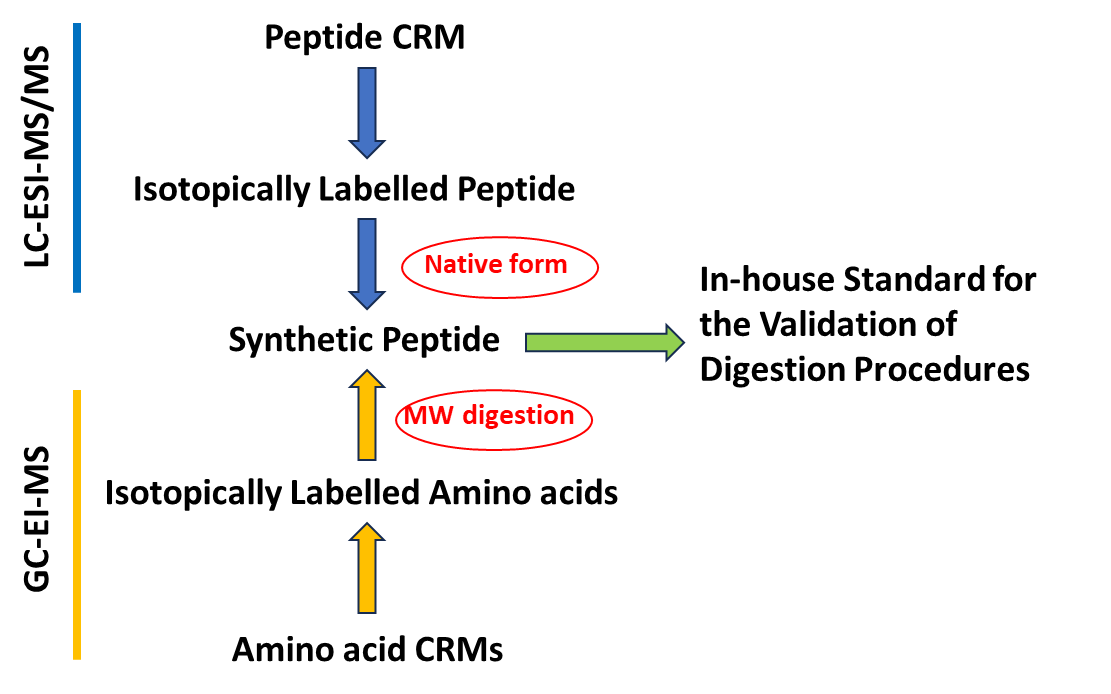


**Figure S3**. Study design carried out in this work

**Table S1**.- Chromatographic and mass spectrometric conditions for the analysis of esterified amino acids and angiotensin I by LC-MS/MS.

| **Liquid chromatograph** | **Agilent Infinity 1290** |
| --- | --- |
| Column | Zorbax Eclipse Plus C18 (50 x 2.1mm x 1.8µm) |
| Flow | 0.4 mL min^-1^ |
| Mobile phase A | H_2_O, 0.1 % formic acid |
| Mobile phase B | ACN, 0.1 % formic acid |
| Separation program | 0 min (5 %B )  1 min (5 %B) 8 min (40 %B) 9 min (60 %B) 10 min (5 %B)  12 min (5 %B) |
| Injection volume | 1 μL |
| Column temperature | 25ºC |
| **Mass spectrometer** | **Agilent 6460** |
| Ionization Source | Electrospray jet stream |
| Ionization mode | Positive |
| Gas temperature | 250 °C |
| Gas flow | 8 mL/min |
| Sheath gas T | 300 °C |
| Sheath gas flow | 10 mL/min |
| Nebulizer pressure | 50 psi |
| Capillary voltage | 2000 V |
| Nozzle voltage | 500 V |
| Collision Energy | 15 eV |

**Table S2**.- Chromatographic and mass spectrometric conditions for the analysis of silanized amino acids by GC-MS.

| **Gas chromatograph** | **Agilent 7879A** |
| --- | --- |
| Column | DB-5MS, 5%phenyl-95%dimethylpolysiloxane (30m x 0.25 mm x 0.25 μm) |
| Helium flow | 2 mL min^-1^ |
| Separation program | 40ºC (5 min)  40 - 105ºC (3.5 min) a 5ºC min^-1^ 105ºC - 120º (3 min) a 5 ºC min^-1^ 120º - 145º a 10 ºC min^-1^ 145º - 185º a 5 ºC min^-1^ 185 - 200º (0.5 min) a 10ºC min^-1^  Total time = 40 min |
| Injection volume | 1 μL |
| Injection temperature | 280ºC |
| Injection mode | Splitless |
| **Mass spectrometer** | **Agilent 7000** |
| Temperature of Transfer Line | 250 ºC |
| Temperature of Ion Source | 230ºC |
| Temperature of quadrupole analyzer | 150ºC |

**Table S3**.- Parameters used for the separation by semipreparative liquid chromatography of the synthesized peptides: natural and isotopically labelled angiotensin I.

| Chromatograph | Agilent Infinity 1260 |
| --- | --- |
| Column | AerisPeptide XB-C18 (Phenomenex) |
| Column dimensions | 250x4.6mm x5µm |
| Temperature | 25 °C |
| Detection wavelenght | 280 nm |
| Injection volume | 100 µL |
| Flow | 2 mL/min |
| Phase A | 0.1% formic acid in water |
| Phase B | ACN, 0.1% formic acid |
| Separation | Time (min) - % B |
|  | 0 8 |
|  | 5 8 |
|  | 24 70 |
|  | 29 70 |
|  | 29.5 8 |
|  | 49.5 8 |

**Table S4**.- Measured transitions, theorical and experimental values of the isotopic abundances of these transitions and standard deviation of the measurements for the reported amino acids obtained from n=5 injections in the LC-MS/MS system.

| **Amino acid** | **MRM Transition**  **Protonated molecules→Product ions** | **Theoretical value** | **Experimental value** | **Standard deviation (n=5)** |
| --- | --- | --- | --- | --- |
| Arginine | 231→214 | 0.8819 | 0.8828 | 0.0013 |
|  | 232→215 | 0.1078 | 0.1070 | 0.0014 |
|  | 233→216 | 0.0097 | 0.0096 | 0.0002 |
|  | 234→217 | 0.0006 | 0.0006 | 0.0001 |
| ^15^N_4_-Arginine* | 235→217 | 0.8916 | 0.8916 | 0.0014 |
|  | 236→218 | 0.0991 | 0.0996 | 0.0011 |
|  | 237→219 | 0.0087 | 0.0083 | 0.0004 |
|  | 238→220 | 0.0006 | 0.0004 | 0.0001 |
| Proline | 172→116 | 0.9385 | 0.9367 | 0.0001 |
|  | 173→117 | 0.0560 | 0.0575 | 0.0001 |
|  | 174→118 | 0.0052 | 0.0055 | 0.0000 |
|  | 175→119 | 0.0002 | 0.0003 | 0.0000 |
| ^13^C_1_-Proline | 172→116 | 0.0000 | 0.0004 | 0.0001 |
|  | 173→117 | 0.9489 | 0.9482 | 0.0003 |
|  | 174→118 | 0.0463 | 0.0464 | 0.0002 |
|  | 175→119 | 0.0048 | 0.0050 | 0.0001 |
| Valine | 174→118 | 0.9383 | 0.9371 | 0.0014 |
|  | 175→119 | 0.0562 | 0.0594 | 0.0016 |
|  | 176→120 | 0.0053 | 0.0035 | 0.0003 |
|  | 177→121 | 0.0002 | 0.0000 | 0.0000 |
| ^13^C_1_-Valine | 174→118 | 0.0012 | 0.0005 | 0.0006 |
|  | 175→119 | 0.9485 | 0.9494 | 0.0013 |
|  | 176→120 | 0.0465 | 0.0454 | 0.0016 |
|  | 177→121 | 0.0048 | 0.0047 | 0.0008 |
| Tyrosine | 238→182 | 0.8966 | 0.8972 | 0.0019 |
|  | 239→183 | 0.0928 | 0.0928 | 0.0016 |
|  | 240→184 | 0.0099 | 0.0096 | 0.0005 |
|  | 241→185 | 0.0007 | 0.0005 | 0.0001 |
| ^13^C_2_-Tyrosine | 238→182 | 0.0001 | 0.0222 | 0.0004 |
|  | 239→183 | 0.0199 | 0.0189 | 0.0006 |
|  | 240→184 | 0.9057 | 0.8815 | 0.0005 |
|  | 241→185 | 0.0742 | 0.0696 | 0.0006 |
| Leucine | 188→132 | 0.9281 | 0.9227 | 0.0013 |
|  | 189→133 | 0.0658 | 0.0698 | 0.0014 |
|  | 190→134 | 0.0058 | 0.0073 | 0.0005 |
|  | 191→135 | 0.0003 | 0.0001 | 0.0001 |
| ^13^C_1_-Leucine | 188→132 | 0.0096 | 0.0002 | 0.0001 |
|  | 189→133 | 0.9293 | 0.9355 | 0.0008 |
|  | 190→134 | 0.0559 | 0.0593 | 0.0007 |
|  | 191→135 | 0.0052 | 0.0050 | 0.0004 |
| Isoleucine | 188→69 | 0.9467 | 0.9472 | 0.0003 |
|  | 189→70 | 0.0522 | 0.0517 | 0.0002 |
|  | 190→71 | 0.0012 | 0.0011 | 0.0000 |
|  | 191→72 | 0.0000 | 0.0000 | 0.0000 |
| ^13^C_1_-Isoleucine** | 189→69 | 0.9467 | 0.9476 | 0.0017 |
|  | 190→70 | 0.0522 | 0.0525 | 0.0015 |
|  | 191→71 | 0.0012 | 0.0011 | 0.0001 |
|  | 192→72 | 0.00001 | 0.00004 | 0.00003 |
| Phenylalanine | 222→166 | 0.8988 | 0.9001 | 0.0007 |
|  | 223→167 | 0.0927 | 0.0917 | 0.0007 |
|  | 224→168 | 0.0080 | 0.0078 | 0.0001 |
|  | 225→169 | 0.0005 | 0.0004 | 0.0001 |
| ^13^C_1_-Phenylalanine | 222→166 | 0.0049 | 0.0055 | 0.0009 |
|  | 223→167 | 0.9054 | 0.9039 | 0.0010 |
|  | 224→168 | 0.0828 | 0.0834 | 0.0008 |
|  | 225→169 | 0.0069 | 0.0071 | 0.0007 |

*Loss of ^15^N_1_

**Loss of ^13^C_1_

**Table S5**.- Measured transitions, theorical and experimental values of the isotopic abundances of these transitions and standard deviation of the measurements for the reported amino acids obtained from n=5 injections in the GC-MS/MS system.

| **Amino acid** | **MRM Transition**  **Protonated molecules→Product ions** | **Theoretical value** | **Experimental value** | **Standard deviation (n=5)** |
| --- | --- | --- | --- | --- |
| Proline | 286 → 258 | 0.7306 | 0.7421 | 0.0003 |
|  | 287 → 259 | 0.1838 | 0.1771 | 0.0006 |
|  | 288 → 260 | 0.0737 | 0.0700 | 0.0006 |
|  | 289 → 261 | 0.0118 | 0.0109 | 0.0001 |
| ^13^C_1_-Proline* | 287 → 258 | 0.7421 | 0.7309 | 0.0007 |
|  | 288 → 259 | 0.1771 | 0.1829 | 0.0006 |
|  | 289 → 260 | 0.0700 | 0.0743 | 0.0003 |
|  | 290 → 261 | 0.0109 | 0.0019 | 0.0001 |
| Valine | 288 → 260 | 0.7419 | 0.7283 | 0.0005 |
|  | 289 → 261 | 0.1772 | 0.1840 | 0.0003 |
|  | 290 → 262 | 0.0700 | 0.0753 | 0.0003 |
|  | 291 → 263 | 0.0109 | 0.0124 | 0.0001 |
| ^13^C_1_-Valine* | 289 → 260 | 0.7419 | 0.7279 | 0.0003 |
|  | 290 → 261 | 0.1772 | 0.1843 | 0.0003 |
|  | 291 → 262 | 0.0700 | 0.0758 | 0.0002 |
|  | 292 → 263 | 0.0109 | 0.0121 | 0.0001 |
| Tyrosine | 466 → 438 | 0.6155 | 0.6072 | 0.0009 |
|  | 467 → 439 | 0.2461 | 0.2481 | 0.0006 |
|  | 468 → 440 | 0.1105 | 0.1154 | 0.0004 |
|  | 469 → 441 | 0.0280 | 0.0293 | 0.0002 |
| ^13^C_2_-Tyrosine | 468 → 440 | 0.6285 | 0.6266 | 0.0010 |
|  | 469 → 441 | 0.2377 | 0.2377 | 0.0007 |
|  | 470 → 442 | 0.1076 | 0.1090 | 0.0008 |
|  | 471 → 443 | 0.0262 | 0.0266 | 0.0003 |
| Leucine | 302 → 274 | 0.7339 | 0.7177 | 0.0010 |
|  | 303 → 275 | 0.1834 | 0.1914 | 0.0004 |
|  | 304 → 276 | 0.0712 | 0.0778 | 0.0005 |
|  | 305 → 277 | 0.0115 | 0.0131 | 0.0001 |
| ^13^C_1_-Leucine* | 303 → 274 | 0.7339 | 0.7185 | 0.0009 |
|  | 304 → 275 | 0.1834 | 0.1907 | 0.0009 |
|  | 305 → 276 | 0.0712 | 0.0777 | 0.0003 |
|  | 306 → 277 | 0.0115 | 0.0131 | 0.0004 |
| Isoleucine | 302 → 274 | 0.7339 | 0.7141 | 0.0006 |
|  | 303 → 275 | 0.1834 | 0.1931 | 0.0010 |
|  | 304 → 276 | 0.0712 | 0.0795 | 0.0005 |
|  | 305 → 277 | 0.0115 | 0.0132 | 0.0003 |
| ^13^C_1_-Isoleucine* | 303 → 274 | 0.7339 | 0.7171 | 0.0019 |
|  | 304 → 275 | 0.1834 | 0.1919 | 0.0014 |
|  | 305 → 276 | 0.0712 | 0.0778 | 0.0004 |
|  | 306 → 277 | 0.0115 | 0.0132 | 0.0002 |
| Phenylalanine | 336 → 308 | 0.7110 | 0.6971 | 0.0007 |
|  | 337 → 309 | 0.2006 | 0.2079 | 0.0003 |
|  | 338 → 310 | 0.0749 | 0.0803 | 0.0006 |
|  | 339 → 311 | 0.0135 | 0.0147 | 0.0003 |
| ^13^C_1_-Phenylalanine* | 337 → 308 | 0.7110 | 0.7064 | 0.0022 |
|  | 338 → 309 | 0.2006 | 0.2038 | 0.0026 |
|  | 339 → 310 | 0.0749 | 0.0760 | 0.0012 |
|  | 340 → 311 | 0.0135 | 0.0137 | 0.0006 |
| Arginine | 213 → 171 | 0.7912 | 0.7734 | 0.0008 |
|  | 214 → 172 | 0.1389 | 0.1487 | 0.0004 |
|  | 215 → 173 | 0.0629 | 0.0699 | 0.0003 |
|  | 216 → 174 | 0.0070 | 0.0080 | 0.0001 |
| ^15^N_4_-Arginine** | 215 → 173 | 0.7962 | 0.7934 | 0.0006 |
|  | 216 → 174 | 0.1350 | 0.1367 | 0.0005 |
|  | 217 → 175 | 0.0622 | 0.0632 | 0.0002 |
|  | 218 → 176 | 0.0066 | 0.0067 | 0.0001 |

*Loss of ^13^C_1_

**Loss of ^15^N_2_

**Table S6**.- Measured transitions, theorical and experimental values of the isotopic abundances for the three transitions measured and standard deviations of the measurements by LC-MS/MS for natural abundance and isotopically labelled angiotensin I.

| **Compound** | **Transition** | **Theoretical value** | **Experimental value** | **Standard deviation (n=5)** |
| --- | --- | --- | --- | --- |
| Angiotensin I | 433→534 | 0.7378 | 0.7311 | 0.0006 |
|  | 433→535 | 0.2154 | 0.2225 | 0.0007 |
|  | 433→536 | 0.0410 | 0.0399 | 0.0005 |
|  | 433→537 | 0.0058 | 0.0065 | 0.0002 |
| ^13^C_1_-Angiotensin I | 433→534 | 0.0057 | 0.0253 | 0.0010 |
|  | 433→535 | 0.7416 | 0.7377 | 0.0042 |
|  | 433→536 | 0.2084 | 0.1983 | 0.0035 |
|  | 433→537 | 0.0389 | 0.0387 | 0.0015 |
| Angiotensin I | 433→647 | 0.6871 | 0.6852 | 0.0013 |
|  | 433→648 | 0.2464 | 0.2519 | 0.0014 |
|  | 433→649 | 0.0556 | 0.0530 | 0.0005 |
|  | 433→650 | 0.0094 | 0.0100 | 0.0002 |
| ^13^C_1_-Angiotensin I | 433→647 | 0.0062 | 0.0075 | 0.0003 |
|  | 433→648 | 0.6904 | 0.7087 | 0.0031 |
|  | 433→649 | 0.2399 | 0.2351 | 0.0029 |
|  | 433→650 | 0.0532 | 0.0487 | 0.0014 |
| Angiotensin I | 433→619 | 0.6962 | 0.6938 | 0.0011 |
|  | 433→620 | 0.2419 | 0.2404 | 0.0009 |
|  | 433→621 | 0.0523 | 0.0504 | 0.0005 |
|  | 433→622 | 0.0084 | 0.0080 | 0.0002 |
| ^13^C_1_-Angiotensin I | 433→619 | 0.0063 | 0.0093 | 0.0003 |
|  | 433→620 | 0.6995 | 0.6978 | 0.0031 |
|  | 433→621 | 0.2352 | 0.2342 | 0.0033 |
|  | 433→622 | 0.0499 | 0.0482 | 0.0001 |

**Figure S4**. Separation of amino acids mixtures at 10 µg·g^-1^ detected in MRM mode: A) LC and B) GC.


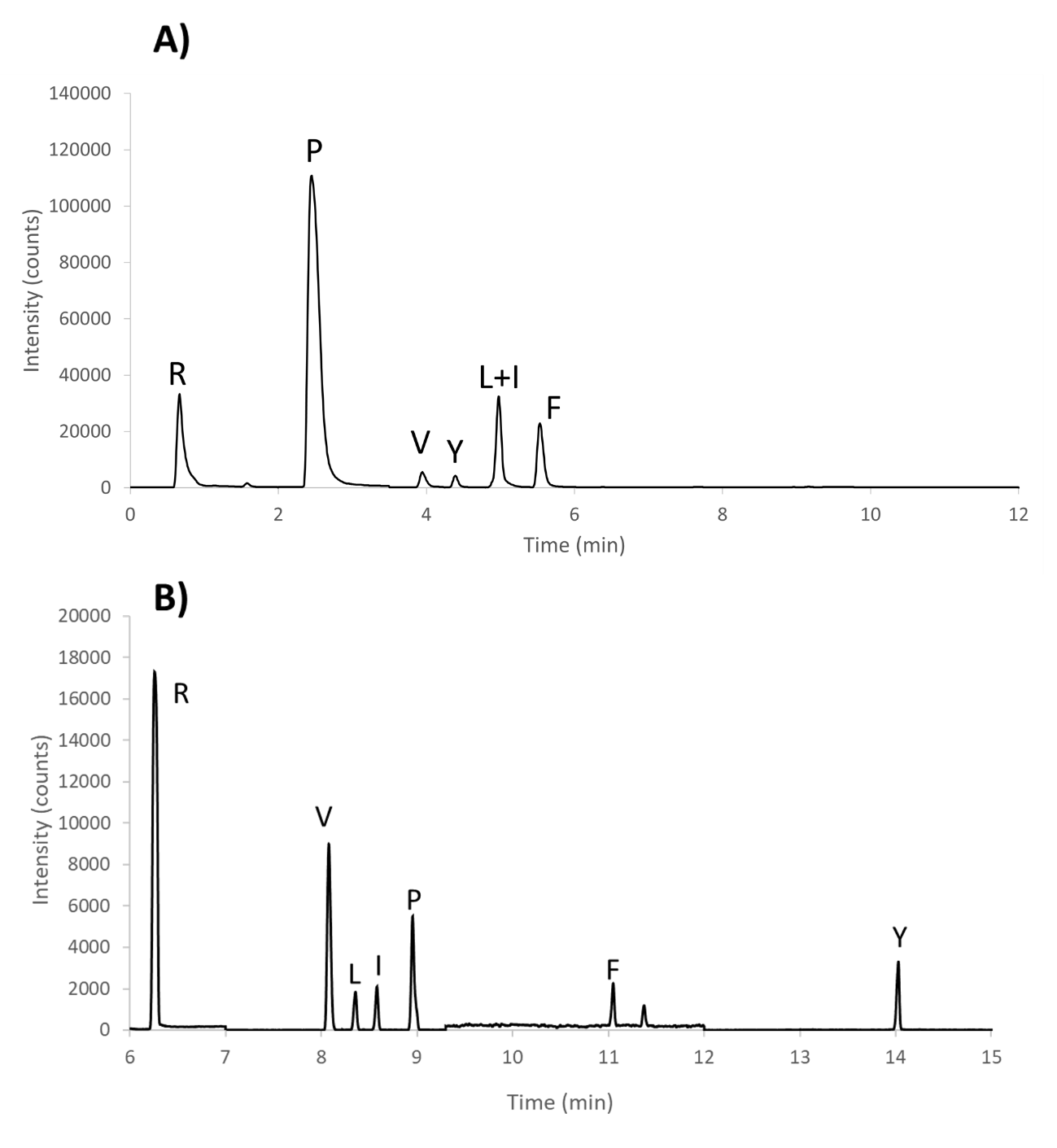


**Figure S5**.- Mean recoveries obtained for the CRM SRM 998 Angiotensin I by classical acid hydrolysis measuring the amino acids cleaved by GC-MS/MS.

**Figure S6**. Mass spectra of the product ions for the precursor ion [M+H_3_]^3+^ at mass 432.8 (natural angiotensin, top) and 433.2 (labelled angiotensin, bottom).


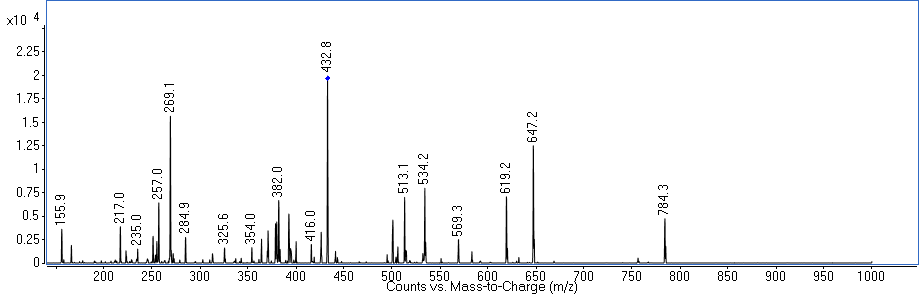

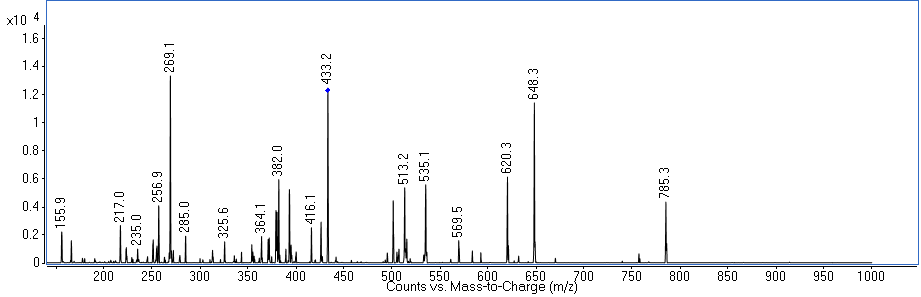


**Figure S7**.- Precursor ion scan of the mixture of natural and isotopically enriched angiotensin for the product ion 647.7, 648.7, 649.7 and 650.7 corresponding to ion b5+ in four transmission conditions: G=20, W=4, B) G=10, W=4, C) G=10, W=2, D) G=10, W=1. (G= gain offset, W= width offset)


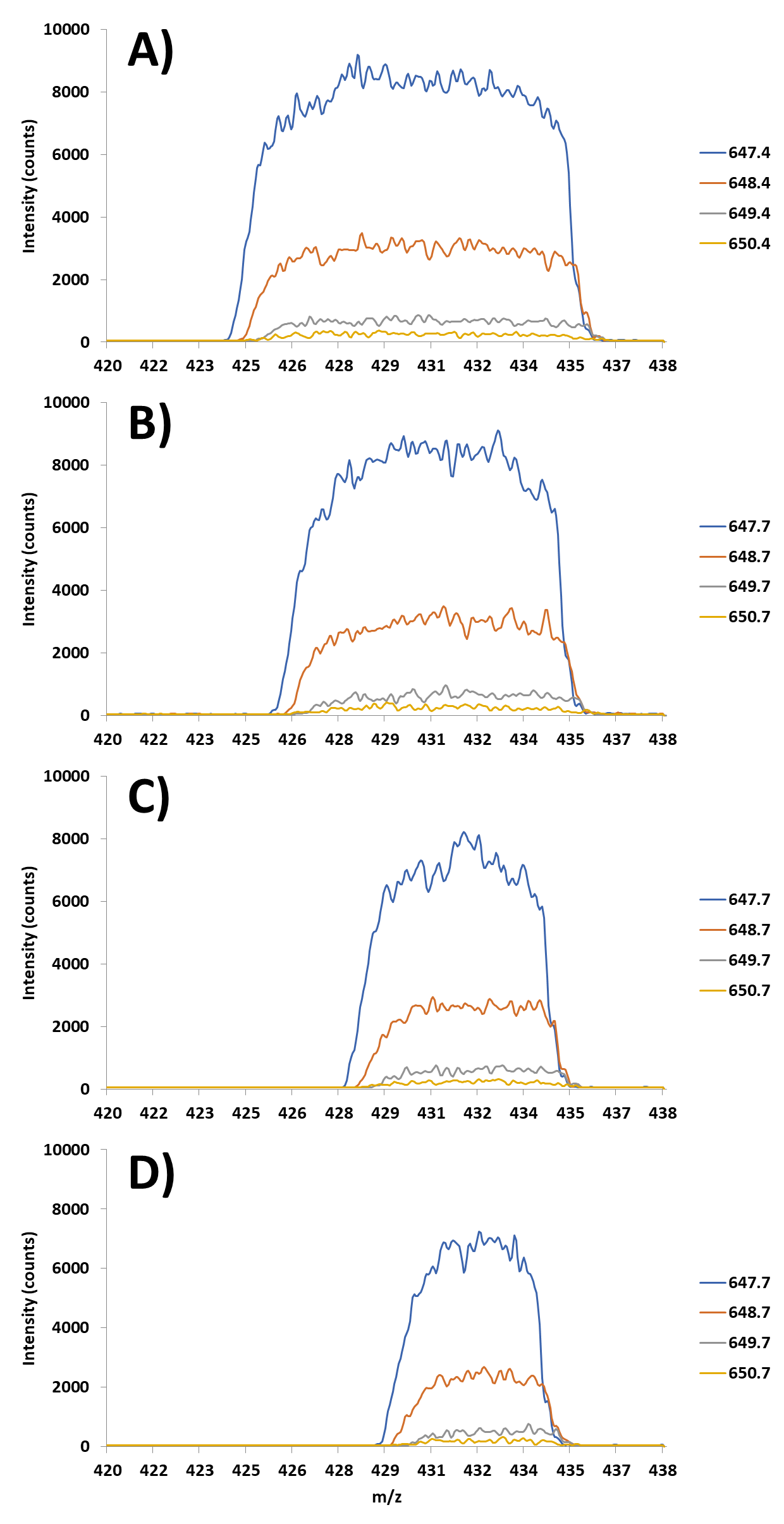

Supplement: Supplementary file 1 — Supplementary file1 (DOCX 506 KB) [file 216_2024_5176_MOESM1_ESM.docx]
